# Interpreting and visualizing pathway analyses using embedding representations with PAVER

**DOI:** 10.6026/973206300200700

**Published:** 2024-07-31

**Authors:** William G Ryan V, Ali Sajid Imami, Hunter Ali Sajid, John Vergis, Xiaolu Zhang, Jarek Meller, Rammohan Shukla, Robert McCullumsmith

**Affiliations:** 1 Department of Neurosciences, College of Medicine and Life Sciences, University of Toledo, Toledo, OH, USA; 2 Department of Microbiology and Immunology, Louisiana State University Health Sciences Center, Shreveport, LA, USA; 3 Department of Environmental and Public Health Sciences, University of Cincinnati, Cincinnati, OH, USA; 4 Department of Computer Science, University of Cincinnati, Cincinnati, OH, USA; 5 Division of Biomedical Informatics, Cincinnati Children's Hospital Medical Center, Cincinnati, OH, USA; 6 Department of Informatics, Nicolaus Copernicus University, Torun, Poland; 7 Department of Zoology & Physiology, College of Agriculture, Life Sciences and Natural Resources, University of Wyoming, Laramie, WY, USA; 8 Neurosciences Institute, ProMedica, Toledo, OH, USA; 9 Department of Psychiatry, College of Medicine and Life Sciences, University of Toledo, Toledo, OH, USA

**Keywords:** Biocuration, computational biology, gene expression profiling, systems biology, pathway analysis

## Abstract

Omics studies use large-scale high-throughput data to explain changes underlying different traits or conditions. However, omics analysis often results in long lists of pathways that are difficult to interpret. Therefore, it is of interest to describe a tool named PAVER (Pathway Analysis Visualization with Embedding Representations) for large scale genomic analysis. PAVER curates similar pathways into groups, identifies the pathway most representative of each group, and provides publication-ready intuitive visualizations. PAVER clusters pathways defined by their vector embedding representations and then identifies the term most cosine similar to its respective cluster's average embedding. PAVER can integrate multiple pathway analyses, highlight relevant biological insights, and work with any pathway database.

## Background:

Multiomics, like transcriptomics, proteomics and kinomics, are used today in experimental biological research to study systems of disease and for precision medicine in clinical settings [1, 2]. The development of these technologies has outpaced researcher's expertise in analyzing data they collect [3]. This "data deluge" exceeds the capacity of human cognition [4, 5]. Analysis of omics is now a leading expense and bottleneck in most projects, limiting its translation from bench-to-bedside [6, 7-8]. Pathway analysis has since become common to interpret high-throughput experiments and explain mechanisms of biological phenomena [9]. However, pathway analysis generally outputs lists of results too long to manually inspect [10, 11]. Various applications have been developed accordingly to summarize information from pathway analyses by selecting most representative terms (MRTs) - the key biological theme defining functionally related groups of pathways - using semantic similarity of Gene Ontology (GO) terms [12, 13, 14, 15-16]. Semantic similarity measures closeness in meaning between GO terms for interpretation, gene clustering and disease-gene prediction [17]. These applications tend to lack complete interoperability beyond controlled ontologies like GO; restricting them from other pathway knowledge bases [18, 19- 20]. The growing volume of omics data indicates a need for novel ways of data management, like automated interpretation of omics results [21, 22].

**Figure 1:**
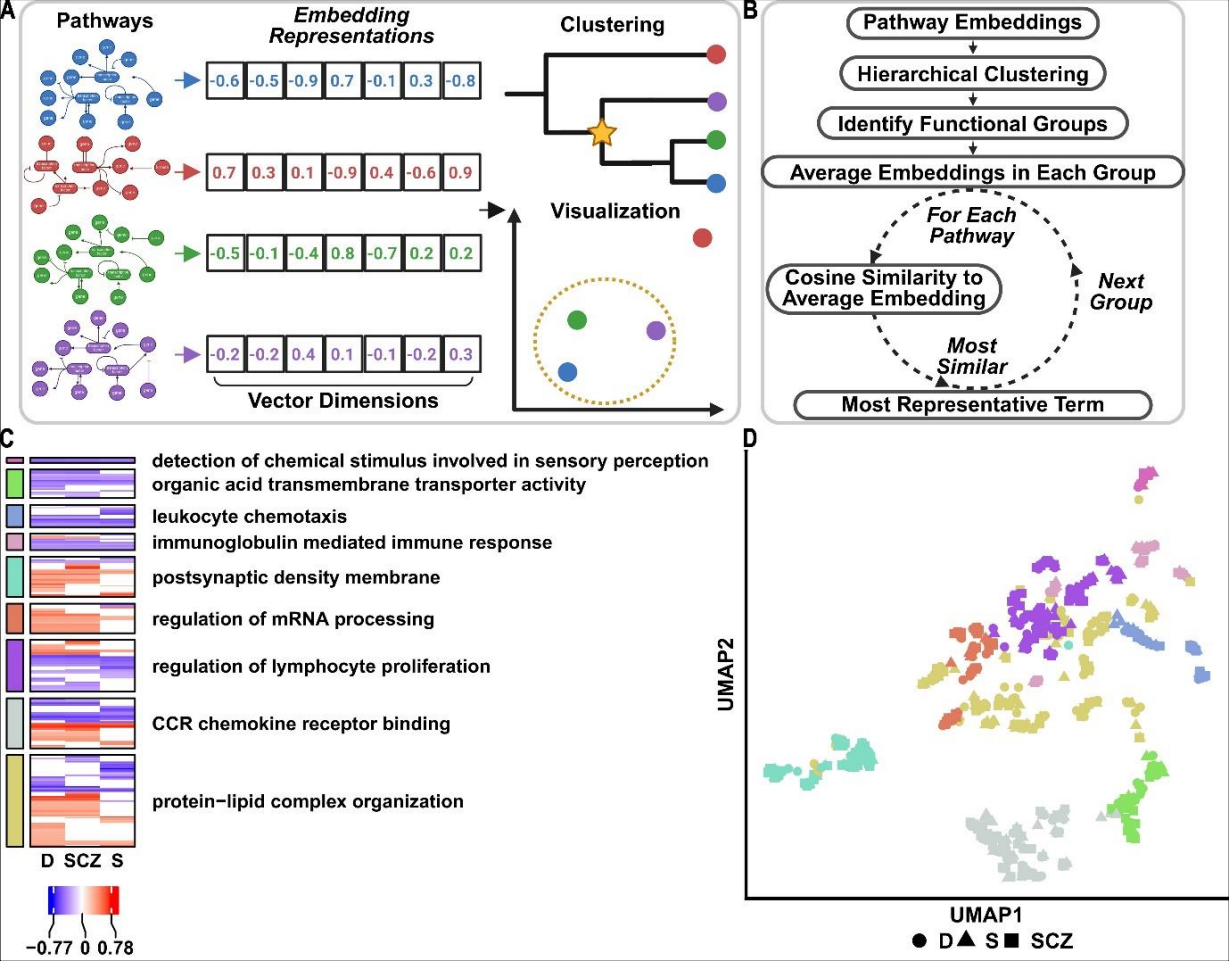
PAVER uses numerical representations of biological pathways to identify functionally related clusters (A) Conceptual overview of the PAVER method implemented in an R programming language software package and web application. Precomputed embedding representations of biological pathways are used for clustering and visualization to aid interpretation of pathway analyses. (B) Diagram of the underlying PAVER algorithm. PAVER is a novel method to select MRTs from groups of functionally related pathways by averaging their embeddings and determining which individual pathway is most cosine similar to its respective group's average. (C) A heatmap generated by the PAVER R package showing uniquely colored-coded clusters of pathways and their identified MRTs from a previously manually interpreted pathway analysis that delineated deep (D), superficial (S), or combined (SCZ) cortical lamina neurons in a bulk RNAseq study of postmortem chronic schizophrenia brain. Legend shows enrichment score from GSEA [40] (D) A scatterplot generated by the PAVER R package showing the 2D computed UMAP of the pathway embeddings. Points show GO terms. Shape indicates respective pathway analysis. Color shows cluster membership for each pathway. MRT: Most Representative Term, GSEA: Gene-set enrichment analysis, 2D: two-dimension, UMAP: Uniform Manifold Approximation and Projection

Modern AI is now being applied to biomarker discovery, survival prognosis and disease subtyping [23, 24]. Recently, embedding models that generate representations of biomedical ontologies have been developed for machine learning techniques like clustering and visualization [25, 26- 27]. Embeddings are numeric vector definitions of pathways that capture their meaning for use as a measure of semantic similarity [26, 28]. This allows for mathematics between words e.g., "Genome - Genes + Proteins = Proteome," where the meaning of different words can be averaged to capture their overall sentiment [29]. On biomedical corpora, embeddings can represent millions of words in hundreds of numerical dimensions [30, 31]. Representing the combined meaning of words with their average embedding in this way has been applied to biological prediction tasks [32]. Embedding models have also been used to define biological entities, like pathways, as the average embedding of their constituent gene members to predict protein-protein interactions [33, 34]. Here, we present PAVER, a novel method that extends this concept by using embedding representations to measure semantic similarity of pathways and identify MRTs in groups of related pathways (Figure 1A). The PAVER algorithm (Figure 1B) first hierarchically clusters pathway embedding's. Pathway embedding's then averaged for each cluster to capture its overall meaning into a single numerical representation. The MRT is finally selected by determining which pathway is most cosine similar to its respective cluster's average embedding. This allows PAVER to curate long lists of pathways into related groups and identify the pathway most representative of each group. PAVER is implemented in a freely available R programming language software package and web application for researchers to integrate, interpret and visualize common pathway analysis outputs.

## Input and Output:

PAVER requires two inputs: pathway analysis results and pre-computed pathway embedding's. Pathway analysis results are expected to be a wide-format table where the first column contains pathway identifiers (e.g. GO: 0005739, hsa04512, WP4562, etc.) and the following columns contain their respective enrichment metrics (e.g. p-value, enrichment score, combined score, etc.) returned from tools like Enrichr or gene-set enrichment analysis [35, 36]. PAVER works generally with any set of pre-computed embedding's. PAVER provides precomputed pathway embedding's using the recent anc2vec embedding model of GO. [25] PAVER also provides precomputed pathway embedding's for GO and Kyoto Encyclopedia of Genes and Genomes (KEGG) using the recent "text-embedding-3-large" embedding model provided by Open AI. These Open AI models have been shown to link biomedical concepts like relationships between diseases, genes and epidemiology [37, 38-39]. We created multi-lined word strings for submission to the embedding model by concatenating each GO term's ID, sub-ontology name, and definition or each KEGG pathway's entry, name, description and class. To demonstrate the utility of PAVER, we applied it to previously manually interpreted pathway analysis results to identify MRTs that delineate deep versus superficial cortical lamina neuron function in a bulk RNAseq study of postmortem chronic schizophrenia brain. [40] PAVER identified MRTs like *detection of chemical stimulus involved in sensory perception*, *postsynaptic density membrane*, and *CCR chemokine receptor binding that closely mirrored manual curation*, like *sensory system*, *synapse*, and *cytokine immunity*, and provided intuitive heat map-based (Figure 1C) and scatterplot-based (Figure 1D) visualizations. Notably, PAVER performed this curation and visualization task more quickly than could be achieved manually without.

## Caveats and Future Development:

PAVER provides a novel method for summarization of biological pathways defined by their embedding representations. However, PAVER assumes the input pathway analysis was properly performed [10]. PAVER also requires that embedding representations are pre-computed. PAVER's proof-of-concept has previously been used in a number of studies to aid in the interpretation of pathway analyses and helps explain mechanisms underlying different disorders and diseases [41, 42, 43, 44, 45-46]. We plan to further increase the utility of PAVER with additional visualizations and pre-computed pathway embedding's for other pathway databases. We hope PAVER will continue to be a valuable resource to help researchers extract actionable insights from their pathway analyses. The PAVER R package is licensed under the GNU General Public License v3.0.

## Declarations:

The authors declare that shinyapps.io is a hosting service provided by the public benefit corporation Posit. Posit has an excellent reputation for ensuring the up time of their hosted applications. Hence, the URL and application are both sustainable in the long term. We have previous experience using this service to host another application which has been available without interruption for more than five years.

Further, University of Toledo IT security policy prevents us from using the utoledo.edu domain to host applications.
